# Saturation-pulse prepared heart-rate independent inversion-recovery (SAPPHIRE) biventricular T1 mapping: inter-field strength, head-to-head comparison of diastolic, systolic and dark-blood measurements

**DOI:** 10.1186/s12880-022-00843-0

**Published:** 2022-07-07

**Authors:** Mashael Alfarih, João B. Augusto, Kristopher D. Knott, Nasri Fatih, M. Praveen Kumar, Redha Boubertakh, Alun D. Hughes, James C. Moon, Sebastian Weingärtner, Gabriella Captur

**Affiliations:** 1 Barts Heart Center, The Cardiovascular Magnetic Resonance Imaging Unit, St Bartholomew’s Hospital, West Smithfield, London, EC1A 7BE UK; 2 Institute of Cardiovascular Science, University College London, Gower Street, London, WC1E 6BT UK; 3 Department of Cardiac Technology, College of Applied Medial Sciences, Imam Abdulrahman Bin Faisal University, Dammam, Saudi Arabia; 4 Department of Pharmacology, Post Graduate Institute of Medical Education and Research, Chandigarh, India; 5 William Harvey Research Institute, Queen Mary University of London, Charterhouse Square, London, UK; 6 UCL MRC Unit for Lifelong Health and Ageing, 33 Bedford Place, London, WC1B 5JU UK; 7 Electrical and Computer Engineering, University of Minnesota, Minneapolis, MN USA; 8 Department of Imaging Physics, Delft University of Technology, Delft, The Netherlands; 9 Cardiology Department, Royal Free Hospital NHS Trust, Pond St, Hampstead, London, NW3 2QG UK

**Keywords:** T1 mapping, Cardiovascular magnetic resonance, SAPPHIRE, MOLLI

## Abstract

**Background:**

To assess the feasibility of biventricular SAPPHIRE T_1_ mapping in vivo across field strengths using diastolic, systolic and dark-blood (DB) approaches.

**Methods:**

10 healthy volunteers underwent same-day non-contrast cardiovascular magnetic resonance at 1.5 Tesla (T) and 3 T. Left and right ventricular (LV, RV) T_1_ mapping was performed in the basal, mid and apical short axis using 4-variants of SAPPHIRE: diastolic, systolic, 0th and 2nd order motion-sensitized DB and conventional modified Look-Locker inversion recovery (MOLLI).

**Results:**

LV global myocardial T_1_ times (1.5 T then 3 T results) were significantly longer by diastolic SAPPHIRE (1283 ± 11|1600 ± 17 ms) than any of the other SAPPHIRE variants: systolic (1239 ± 9|1595 ± 13 ms), 0th order DB (1241 ± 10|1596 ± 12) and 2nd order DB (1251 ± 11|1560 ± 20 ms, all *p* < 0.05). In the mid septum MOLLI and diastolic SAPPHIRE exhibited significant T_1_ signal contamination (longer T_1_) at the blood-myocardial interface not seen with the other 3 SAPPHIRE variants (all *p* < 0.025). Additionally, systolic, 0th order and 2nd order DB SAPPHIRE showed narrower dispersion of myocardial T_1_ times across the mid septum when compared to diastolic SAPPHIRE (interquartile ranges respectively: 25 ms, 71 ms, 73 ms *vs* 143 ms, all *p* < 0.05). RV T_1_ mapping was achievable using systolic, 0th and 2nd order DB SAPPHIRE but not with MOLLI or diastolic SAPPHIRE. All 4 SAPPHIRE variants showed excellent re-read reproducibility (intraclass correlation coefficients 0.953 to 0.996).

**Conclusion:**

These small-scale preliminary healthy volunteer data suggest that DB SAPPHIRE has the potential to reduce partial volume effects at the blood-myocardial interface, and that systolic SAPPHIRE could be a feasible solution for right ventricular T_1_ mapping. Further work is needed to understand the robustness of these sequences and their potential clinical utility.

**Supplementary Information:**

The online version contains supplementary material available at 10.1186/s12880-022-00843-0.

## Background

**Fig. 1:**
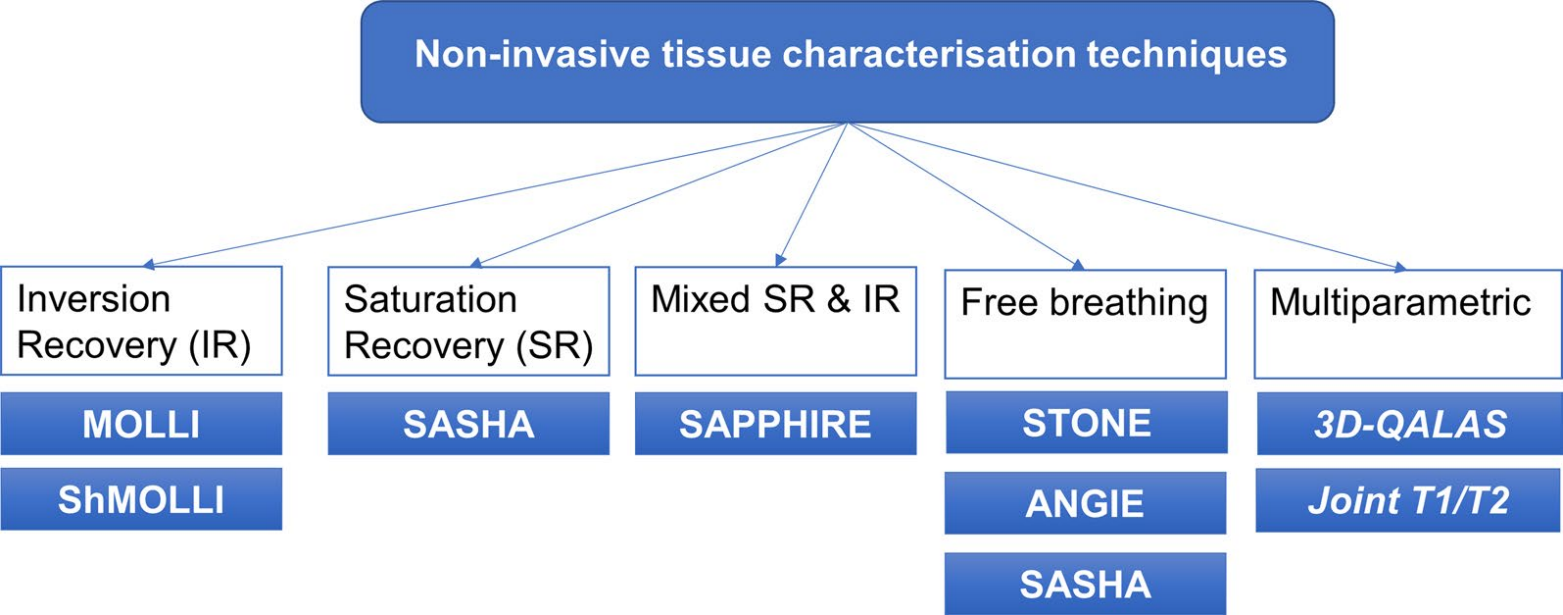
Basic overview of T1 mapping acquisition strategies. ANGIE, Accelerated and Navigator-Gated Look-Locker Imaging for Cardiac T1 Estimation; 3D-QALAS, three-dimensional-QuAntification using an interleaved Look-Locker Acquisition Sequence with T2 preparation pulse; MOLLI, Modified Look-Locker Inversion recovery; Prep, preparation; SAPPHIRE, Saturation Pulse Prepared Heart-Rate Independent Inversion REcovery Sequence; SASHA, saturation recovery single shot acquisition; SAT, saturation; Seg, segmented; ShMOLLI, shortened MOLLI; STONE, slice-interleaved T1 mapping sequence (Radenkovic D, 2017)

The myocardial longitudinal relaxation time, T_1_ is a sensitive imaging biomarker for heart muscle disease, linked to both functional capacity and mortality [1–5]. Various techniques have been proposed to quantify T_1_ relaxation (Fig. 1), each with its own advantages and limitations [6]. One of the main issues of commonly-used T_1_ mapping techniques is the partial volume effect that can artifactually increase the myocardial T_1_ times of voxels located at the myocardial-blood pool interface due to confounding by the higher native T_1_ signal of the blood pool. This is most apparent in cross sectional imaging and its effects are especially detrimental to the study of thin-walled cardiac structures, notably the right ventricular (RV) free wall, atrial walls and the thinned myocardium in dilated cardiomyopathy [7, 8]. Partial volume effects are problematic because they reduce the accuracy and reproducibility of T_1_ mapping. To overcome this problem in the right and left ventricles (LV), systolic readouts have been proposed [9, 10], as it was hypothesized that the increased myocardial wall thickness during systole would increase the abundance of myocardial voxels that were free from partial volume effects. Systolic readouts collect data during a limited quiescent time window and therefore necessitate a shorter acquisition time to reduce temporal blurring, however this trade off could affect precision. In spite of this limitation, systolic T1 mapping approaches have potential advantages in arrhythmia and resulted in more evaluable images [9–11].

The modified Look-Locker inversion recovery (MOLLI) sequence is still the most commonly used T_1_ mapping approach in clinical practice due to its widespread availability across vendors and superior precision and reproducibility [12]. Being an inversion recovery-based approach, it is vulnerable to the aforementioned partial volume effects, as well as to off-resonance artifacts, and confounding contributions by magnetisation transfer and T2 effects [11].

A T_1_ mapping sequence that combines saturation recovery and inversion recovery approaches, termed SAPPHIRE–SAturation Pulse Prepared Heart-rate independent Inversion REcovery–has recently been shown to have potential advantages in arrhythmia [13]. A further refinement to SAPPHIRE that provides blood suppression resulting in dark-blood (DB) native myocardial T_1_ mapping has shown additional promise at addressing the problem of myocardial signal contamination from the adjacent blood pool [8]. DB SAPPHIRE potentially measures more accurate T_1_ than the conventional diastolic SAPPHIRE because of less contamination from the blood pool signal, but this is at the expense of precision.

Here we undertake a head-to-head comparison of SAPPHIRE T_1_ mapping variants across field strengths. We assess the performance of LV and RV SAPPHIRE T_1_ mapping in vivo considering 4 variants: diastolic, systolic, 0th and 2nd order DB SAPPHIRE, and compare results to those obtained by conventional MOLLI across field strengths.

## Methods

All subjects provided written informed consent. The study received ethical approval from the University College London (UCL) Research Ethics Committee (Project number 6782/001) and it conformed to the principles of the Helsinki Declaration.

### Study population and data collection

In this prospective single-center observational study, 10 healthy volunteers underwent non-contrast cardiovascular magnetic resonance (CMR) scanning at both field strengths on the same day at the UCL Bloomsbury Center for Clinical Phenotyping (London, UK). Volunteers had no prior cardiac history or known cardiac risk factors, were not on cardiovascular medications, had a normal resting electrocardiogram (ECG), and were free of conventional contra-indications for CMR.

### Cardiovascular magnetic resonance

CMR studies were performed using two Siemens MR systems (Erlangen, Germany): MAGNETOM AERA 1.5 Tesla (T) operating VE11C-SP01 and MAGNETOM PRISMA 3 T operating VE11C-SP01, with 18-channel phased-array chest coils. The scan protocol was identical for the two field strengths and consisted of localizers, transaxial black blood HASTE anatomical stack, breath-held retrospectively ECG-gated balanced steady-state free precession (bSSFP) cines in standard long and short axis views [14], and then breath-held ECG-gated T_1_ mapping in the basal, mid and apical LV short axis slices (co-registered with the cines) using MOLLI with motion correction (MOCO), bright-blood diastolic SAPPHIRE, bright-blood systolic SAPPHIRE, 0th order DB SAPPHIRE and 2nd order DB SAPPHIRE in random order per scan. To minimize off-resonance artifacts with higher field strengths, the 3 T protocol additionally included frequency scouts and attention to volumetric shimming.

Typical imaging parameters for the T1 mapping sequences are summarised in Table 1.

**Table 1:** Typical imaging parameters for the SAPPHRE sequences

		TR/TE (ms)	TD (ms)	FA	TI (ms)	Matrix	Slice thickness (mm)	FOV (mm)	Pixel size (mm)	Aquisition window (ms)
MOLLI	1.5 T	291.84/1.22	605	35°	~ 103–4035	256 × 192	8	300 × 225	1.171875 × 1.171875	4
	3 T	283.8/1.16	780	20°	~ 103–4438	256 × 192	8	300 × 225	1.171875 × 1.171875	4
Diastolic SAPPHIRE	1.5 T	830/1.12	710	70°	~ 715	256 × 192	8	300 × 225	1.171875 × 1.171875	8
	3 T	856/1.12	778	68°	~ 740	256 × 192	8	300 × 225	1.171875 × 1.171875	8
Systolic SAPPHIRE	1.5 T	830/1.12	315	70^o^	~ 335	256 × 192	8	300 × 225	1.171875 × 1.171875	9
	3 T	857/1.12	393	68^o^	~ 380	256 × 192	8	300 × 225	1.171875 × 1.171875	9
DB 0th order SAPPHIRE^┼^	1.5 T	800/1.28	678	70^o^	~ 670	256 × 192	8	300 × 225	1.171875 × 1.171875	7
	3 T	857/1.12	753	68	~ 740	256 × 192	8	300 × 225	1.171875 × 1.171875	7
DB 2nd order SAPPHIRE^┼^	1.5 T	800/1.28	678	70^o^	~ 670	256 × 192	8	300 × 225	1.171875 × 1.171875	8
	3 T	857/1.12	750	68^o^	~ 740	256 × 192	8	300 × 225	1.171875 × 1.171875	8

DB, Dark blood; FA, flip angle; FOV, Field of view; MOLLI, Modified Look-Locker inversion recovery; SAPPHIRE, SAturation Pulse Prepared Heart-rate independent Inversion Recovery; T, Tesla; TD trigger delay; TI inversion time; TR/TE, time between two consecutive excitations/ echo time

10 images acquired with 10 s breath-hold duration and 125 encoding steps for all sequences

┼MSDE gradient amplitude 20 mT/m with preparation duration 10 ms

### Cardiovascular magnetic resonance analysis

Images were analysed using CVI42 software (Circle Cardiovascular Imaging Inc. v.5.10.1, Calgary, Canada). Measurements were performed by two readers with over three years of advanced cardiac MRI experience (M.A., J.A.). LV volumes, LV ejection fraction (LVEF) and mass were determined according to standardized CMR methods [15] using a semiautomated threshold-based technique and body surface area (BSA) indexation where appropriate. Atrial volumes, LV maximal wall thickness, and mitral and tricuspid annular plane systolic excursions were determined as previously described [16–18].

For native T_1_ measurements in the LV, endo- and epicardial borders were manually drawn per segment according to the 16-segment American Heart Association model, in the 3 short axis slices (10% offset). Segmental T_1_ times were averaged to obtain the mean slice T_1_ and global T_1_ calculated as the mean of basal, mid and apical LV short axis slices.

**Fig. 2:**
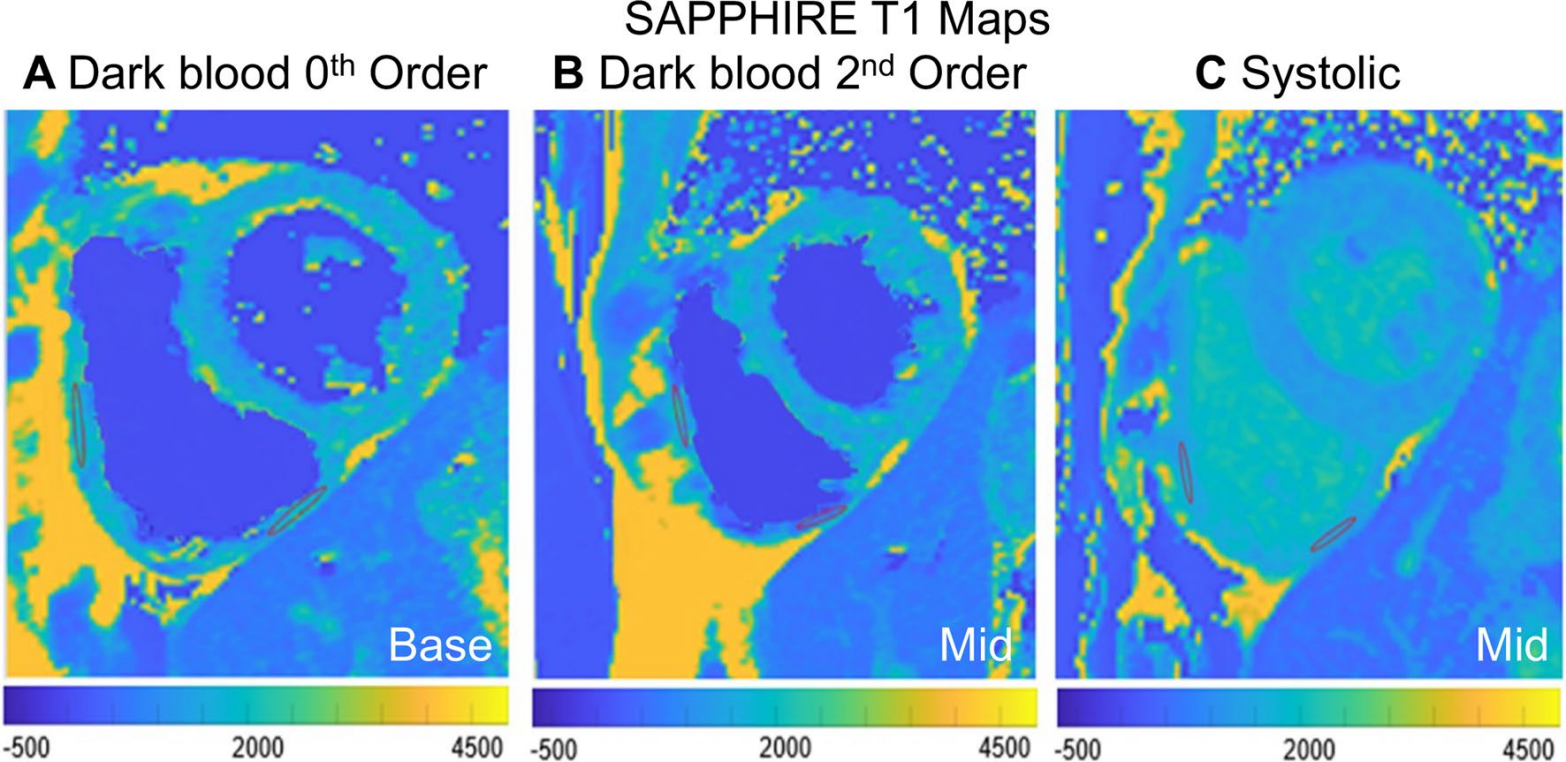
Right ventricular (RV) T_1_ times were determined by manually tracing regions of interest in the RV inferior wall or RV free wall on the basal (**a**) or mid-ventricular (**b**, **c**) short axis dark blood or systolic SAPPHIRE T_1_ map. Exemplar RV ROIs at 1.5 T are shown for 3 study participants. Other abbreviations as in Fig. 1

As demonstrated in Fig. 2 the RV region of interest (ROI) measurements were obtained from the RV inferior wall or free wall in the basal or mid short axis slices if wall thickness ≥ 5 mm.

The transeptal myocardial T_1_ times for MOLLI and SAPPHIRE variants were calculated in OsiriX MD using six evenly-spaced points along linear callipers transecting the mid-septal short axis slice on the T_1_ maps of each healthy volunteer.

Intra- and inter-observer re-read variability was determined for measurements of average mid slice T_1_, and mid septal ROI T_1_ in 5 randomly chosen CMR scans. Intra-observer variability was performed with one-month temporal interval between repeat analyses.

### Native SAPPHIRE T_1_ mapping sequences

#### Diastolic SAPPHIRE

**Fig. 3:**
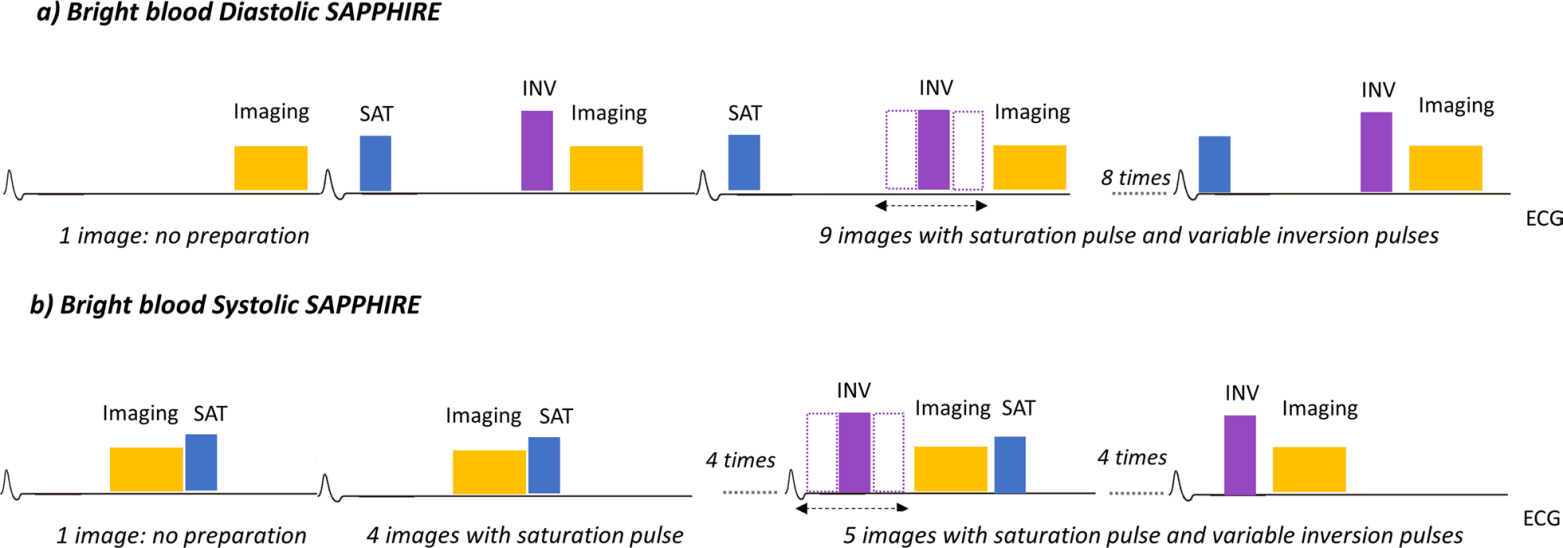
Sequence diagrams of the SAPPHIRE bright blood diastolic (**a**) and systolic (**b**) T_1_ mapping variants. The image acquisition window in systolic SAPPHIRE is shorter compared to diastole. ECG, Electrocardiogram; INV, Inversion pulse. Other abbreviations as in Fig. 1

Conventional diastolic SAPPHIRE (without any attempt to suppress the blood signal, i.e. ‘bright blood’) consists of a combination of saturation and inversion pulses. A saturation pulse is applied immediately after the R wave followed by an inversion pulse inserted in the same heartbeat prior to image acquisition (Fig. 3a). The first image acquisition is done without magnetisation preparation.

#### Systolic SAPPHIRE

Systolic SAPPHIRE also consists of saturation and inversion recovery magnetisation preparation hybrid and 10-ECG triggered readouts but data is acquired during systole, and with shorter acquisition windows than the diastolic SAPPHIRE. Imaging in systole is challenging due to the short time window available between R waves resulting in a weak saturation recovery T_1_ mapping signal. As a fix, saturation is performed in the preceding heart-beat directly following the imaging pulses played in the previous heart-beat. Similar to diastolic SAPPHIRE, the first image is acquired without magnetisation preparation. The remaining images are obtained with an extra inversion pulse with variable delay following the R wave (Fig. 3b).

#### 0th and 2nd Order dark blood diastolic SAPPHIRE

**Fig. 4:**
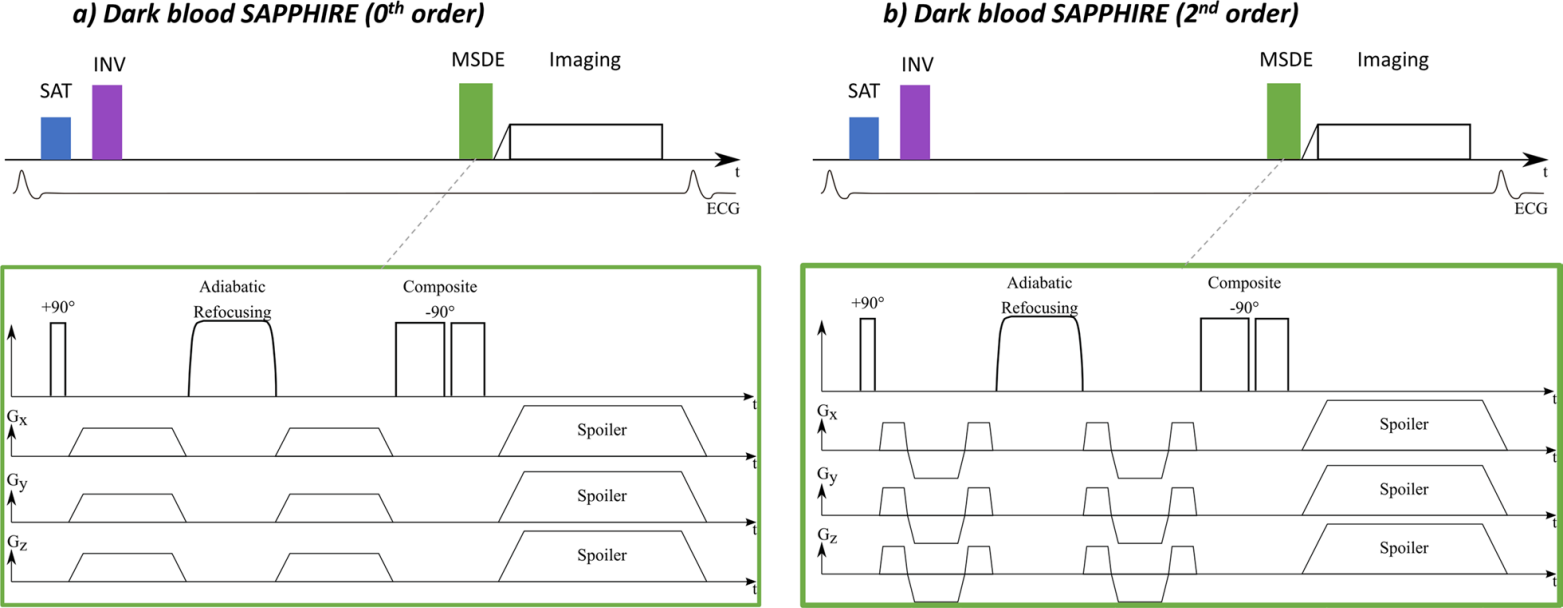
Sequence diagrams of the SAPPHIRE dark blood T 1 mapping variants with 0th (**a**) and 2nd (**b**) order flow sensizting gradients. MSDE, Motion-sensitized driven equilibrium. Other abbreviations as in Figs. 1 and 3

DB T_1_ mapping is achieved using a modified SAPPHIRE technique [19]. For blood suppression, motion sensitized driven equilibrium (MSDE) preparation is inserted before the bSSFP imaging readout (Fig. 4a). The MSDE preparation consists of a 90° excitation tip-down pulse, a one or more 180° refocusing pulse, and a –90° flip-back pulse to encode the spin dephasing in the longitudinal magnetisation (Fig. 4a). Strong motion sensitising gradients are also sandwiched in between the radiofrequency pulses to induce dephasing. Two kinds of motion sensitising gradients are employed, with nulling the gradient moment up to the 0th and 2nd order respectively. To achieve 0th order gradient nulling, identical trapezoidial gradients are played before and after the refocusing pulse.

Additionally, in this study advanced motion sensitising gradients were introduced. As illustrated in Fig. 4b, reverse bipolar gradient blips are inserted to achieve 0th and 2nd moment nulling.

### Data analysis and statistics

Statistical analysis was performed in R programming language (version 3.6.0, The R Foundation for Statistical Computing) and SPSS statistical software (version 26.0, IBM Corp., Armonk, NY, USA). Descriptive data are expressed as mean ± standard deviation except where otherwise stated. The distribution of data was evaluated by histograms and Shapiro–Wilk test. Parametric and nonparametric continuous variables pertaining to participants were compared using student *t*-test or Mann–Whitney U test as appropriate. Categorical variables were compared by χ^2^ or Fisher’s exact tests.

Linear mixed effect models were used to compare T_1_ times across SAPPHIRE techniques (fixed effects: e.g. systolic *vs.* diastolic, 1.5 T *vs.* 3 T, slice level) accounting for repeatedness (random effect: subject ID). In addition, paired-samples *t*-test (parametric) or related-samples Wilcoxon signed rank test (non-parametric) were used for pairwise comparisons between MOLLI and SAPPHIRE, and within SAPPHIRE, across field strengths using Bonferroni correction.

Differences in transeptal T_1_ mapping profiles between sequences were assessed using two samples Anderson–Darling test as it gives more weight to the tails of the distribution (that is close to the blood-myocardial interface which was of particular interest to us). Two-sided *p*-values < 0.05 were considered significant.

Intra- and inter-observer variability (absolute agreement) of T_1_ mapping times were assessed using two-way random, single measures intraclass correlation coefficient (ICC).

## Results

### Study population characteristics

Demographic and CMR characteristics of the 10 healthy volunteers (8 females, age 36 ± 10 years) are presented in Table 2.

**Table 2:** Demographic and CMR characteristics of the healthy volunteers

Healthy volunteers (*n* = 10)	
**Demographics**	
Age, years	36 ± 10
Female	8 (80%)
Height, cm	168.4 ± 8.7
Weight, kg	71.6 ± 13.3
BSA, m^2^	1.8 ± 0.2
**CMR parameters**	
LAVi, mL/m^2^	11.0 ± 2.1
RAVi, mL/m^2^	11.3 ± 1.3
LVEDVi, mL/m^2^	74.6 ± 0.2
LVESVi, mL/m^2^	27.6 ± 0.2
LVEF, %	63 ± 5.1
MAPSE, mm	15.3 ± 2.7
LV MWT, mm	7.8 ± 1.0
LVMi, g/m^2^	48.3 ± 12.5
RVEDVi, mL/m^2^	75.1 ± 9.0
RVESVi, mL/m^2^	38.6 ± 9.9
RVEF, %	49 ± 10.2
RVMi, g/m^2^	25.8 ± 4
TAPSE, mm	25.3 ± 3.1

Data reported as mean ± 1SD or count (%) as appropriate

BSA, body surface area; CMR, cardiovascular magnetic resonance; LAVi, left atrial volume; LVEDVi, left ventricular end-diastolic volume indexed to body surface area; LVEF, left ventricular ejection fraction; LVESVi, left ventricular end-systolic volume indexed to body surface area; LVMi, left ventricular mass; MAPSE, mitral annular plane systolic excursion; MWT, maximum wall thickness; RAVi, right atrial volume; RVEDVi, right ventricular end-diastolic volume indexed to body surface area; RVEF, right ventricular ejection fraction; RVESVi, right ventricular end-systolic volume indexed to body surface area; SD, standard deviation; TAPSE, tricuspid annular plane systolic excursion

### Feasibility in vivo

SAPPHIRE T_1_ mapping using all 4 variants was completed successfully in all subjects (Tables 3, 4), Each SAPPHIRE T_1_ map was acquired within a standard single breathold not dissimilar from a convetional MOLLI acquisition. ROI placement for RV T_1_ mapping was not feasible using MOLLI or diastolic SAPPHIRE because of insufficient wall thickness, but it was possible using systolic, 0th order and 2nd order DB SAPPHIRE and results are reported in Table 4

**Table 3:** Summary of LV T_1_ mapping data by MOLLI and the 4 SAPPHIRE variants across field strengths

Sequence	Field Strength	Basal SAX	Mid SAX	Apical SAX	Mid Septal ROI	Global T_1_	Non-apical Global T_1_
MOLLI*	1.5 T	1032 ± 23	1029 ± 26	1030 ± 21	1037 ± 28	1034 ± 7	1031 ± 2
	3 T	1297 ± 27	1297 ± 26	1311 ± 32	1310 ± 36	1298 ± 9	1295 ± 3
Diastolic SAPPHIRE	1.5 T	1261 ± 45	1267 ± 33	1342 ± 60	1250 ± 79	1284 ± 11	1264 ± 3
	3 T	1637 ± 83	1596 ± 48	1593 ± 70	1603 ± 53	1601 ± 17	1612 ± 6
Systolic SAPPHIRE	1.5 T	1286 ± 51	1225 ± 21	1239 ± 18	1225 ± 42	1239 ± 9	1253 ± 5
	3 T	1580 ± 41	1586 ± 44	1596 ± 43	1585 ± 48	1596 ± 13	1591 ± 4
DB 0th order SAPPHIRE	1.5 T	1229 ± 57	1248 ± 30	1246 ± 55	1228 ± 78	1241 ± 10	1242 ± 3
	3 T	1571 ± 69	1595 ± 46	1608 ± 42	1597 ± 74	1596 ± 12	1584 ± 5
DB 2nd order SAPPHIRE	1.5 T	1235 ± 46	1225 ± 61	1298 ± 51	1220 ± 111	1251 ± 11	1237 ± 5
	3 T	1550 ± 65	1555 ± 64	1569 ± 31	1503 ± 132	1561 ± 20	1561 ± 4

Values reported are mean ± 1SD

*At our CMR Unit (the UCL Bloomsbury Center for Clinical Phenotyping) normal values for native myocardial T_1_ by MOLLI in healthy volunteers are 1030 ± 32 ms at 1.5 T and 1280 ± 46 ms at 3 T

DB, Dark blood; MOLLI, Modified Look-Locker inversion recovery; ROI, region of interest; SAPPHIRE, SAturation Pulse Prepared Heart-rate independent Inversion REcovery; SAX, short axis; T, Tesla. Other abbreviations as in Table 1

**Table 4:** Summary of RV T_1_ mapping times by MOLLI and the 4 SAPPHIRE variants across field strengths

Sequence	Field Strength	Basal SAX (inferior or free wall)	Mid SAX (inferior wall)	Mid SAX (free wall)	Global T1 ROI
MOLLI*	1.5 T	NA	NA	NA	NA
	3 T	NA	NA	NA	NA
Diastolic SAPPHIRE*	1.5 T	NA	NA	NA	NA
	3 T	NA	NA	NA	NA
Systolic SAPPHIRE	1.5 T	1531 ± 94	1479 ± 98	1422 ± 58	1477 ± 83
	3 T	1766 ± 14	1757 ± 51	1769 ± 31	1764 ± 32
DB 0th order SAPPHIRE	1.5 T	1440 ± 67	1453 ± 122	1453 ± 104	1448 ± 98
	3 T	1724 ± 37	1681 ± 43	1689 ± 49	1698 ± 43
DB 2nd order SAPPHIRE	1.5 T	1524 ± 44	1417 ± 82	1452 ± 54	1464 ± 60
	3 T	1684 ± 101	1667 ± 63	1658 ± 78	1670 ± 81

Values reported are mean ± 1SD

*RV measurements were obtained only when the RV wall thickness ≥ 5 mm and this was not achievable with MOLLI or diastolic SAPPHIRE

DB, Dark blood; MOLLI, Modified Look-Locker inversion recovery; ROI, region of interest; RV, right ventricle; SAPPHIRE, SAturation Pulse Prepared Heart-rate independent Inversion REcovery; SAX, short axis; T, Tesla. Other abbreviations as in Table 1

### Inter-field strength differences

**Fig. 5:**
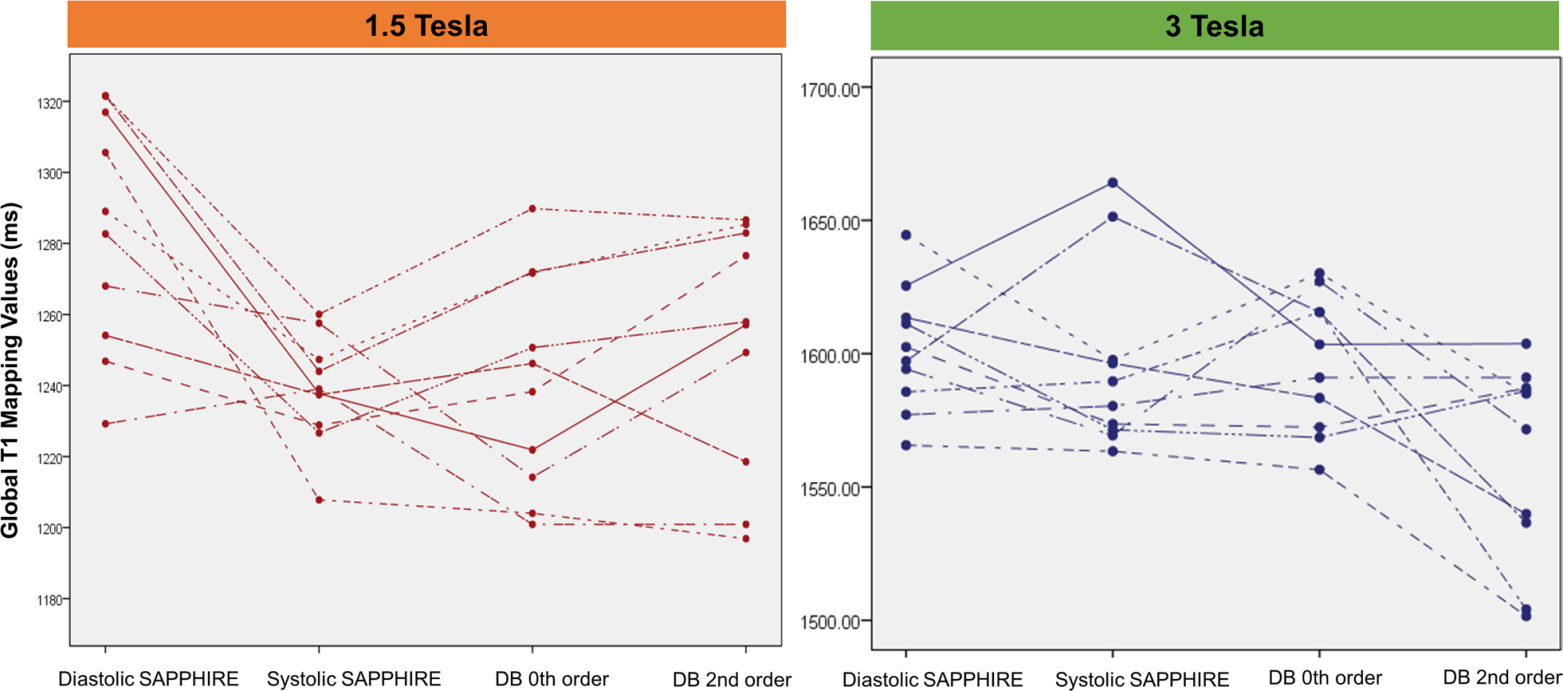
Profile plot displays the global T_1_ times obtained using the various SAPPHIRE variants in all ten healthy volunteers in our study. Each dot represents an individual. DB, Dark blood. Other abbreviations as in Fig. 1

**Fig. 6:**
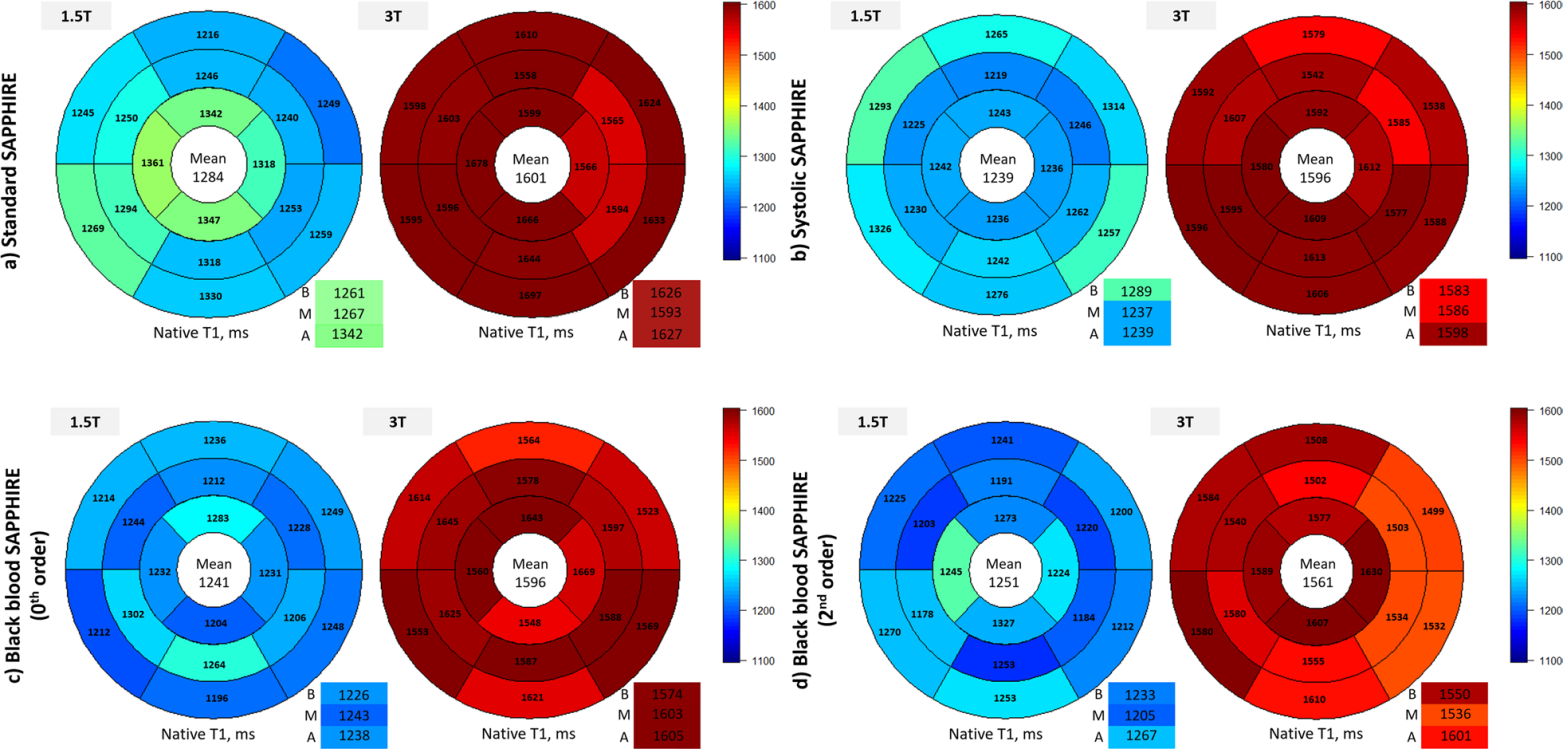
Bullseye plots illustrating the mean segmental T_1_ times of all healthy volunteers in three short-axis slices (B, basal; M, mid-ventricular; A, apical) according to SAPPHIRE variant and field strength (1.5 T blue, 3 T red). Global T_1_ is reported in the center of the bullseye and slice-specific means in the bottom right boxes. T, Tesla. Other abbreviations as in Figs. 1 and 5

Healthy volunteer native myocardial T_1_ times obtained by the 4 SAPPHIRE variants and by MOLLI across field strengths are reported in Table 3, and global LV T_1_ times provided in Fig. 5. As expected, for each sequence T_1_ times were consistently higher at 3 T than at 1.5 T (all *p* < 0.005, Fig. 6).

### Differences between SAPPHIRE variants and MOLLI

Native myocardial T_1_ times by MOLLI for study members at either field strength matched the normal values by this sequence previously established at our center (reported in Table 3). As expected, from the known higher accuracy of saturation-based *vs.* inversion recovery-based T_1_ mapping sequences [20], the 4 SAPPHIRE variants measured longer global myocardial T_1_ times than MOLLI (pairwise comparisons all *p* < 0.005, Additional file 1: Table S1*).* These differences persisted after removing the apical slice data (Additional file 1: Table S2, *p* < 0.005).

### Differences between SAPPHIRE variants

**Fig. 7:**
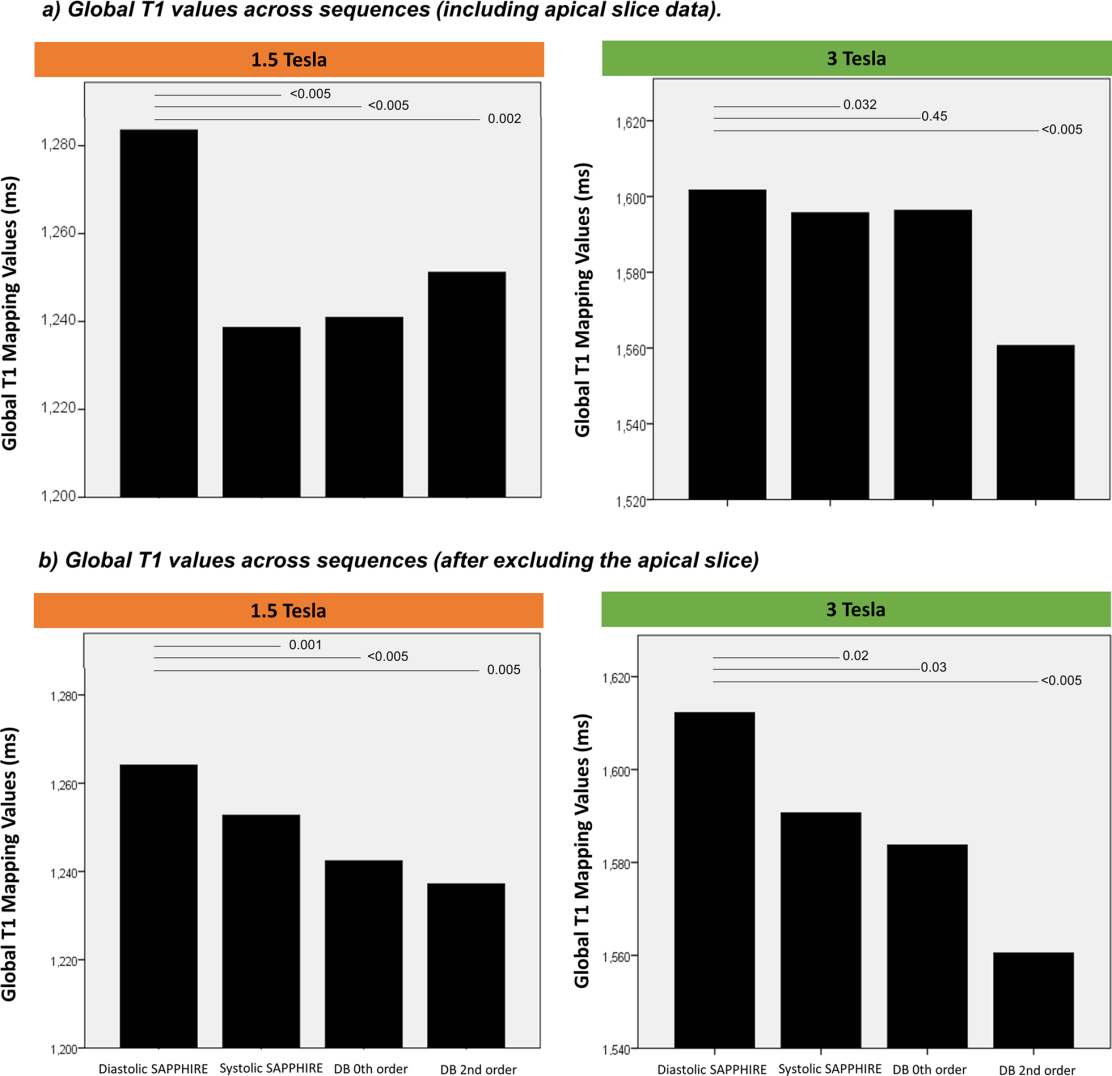
Global T_1_ mapping times at 1.5 T (left) and 3 T (right) by diastolic, systolic, 0th order and 2nd order SAPPHIRE sequences (**a**) including the apical slice (**b**) excluding the apical slice. Numbered lines above the bars represent *p* values for differences obtained using mixed effect models. Abbreviations as in Figs. 1, 5 and 6

Using linear mixed models to adjust for field strength, phase, slice location and subject, myocardial T_1_ times by diastolic SAPPHIRE were significantly longer than with systolic, 0th order DB and 2nd order DB SAPPHIRE at 1.5 T and significantly longer than with systolic and 2nd order DB SAPPHIRE at 3 T (all *p* < 0.05, Fig. 7a). After excluding the apical slice the myocardial T_1_ times by diastolic SAPPHIRE were significantly longer than with systolic, 0th order DB and 2nd order DB SAPPHIRE at both field strengths (all *p* < 0.05, Fig. 7b).

At 1.5 T pairwise comparisons for LV global T_1_ showed that diastolic SAPPHIRE measured significantly longer T_1_ times than the 0th order DB SAPPHIRE (*p* = 0.014, Additional file 1: Table S1) but not after excluding the apical slice data (Additional file 1: Table S2). Pairwise comparisons of RV T_1_ times showed no differences between DB SAPPHIRE variants (all *p* > 0.005, Additional file 1: Table S3). At 3 T systolic SAPPHIRE measured significantly longer RV T_1_ times than the 0th order and 2nd order SAPPHIRE (*p* = 0.028 and 0.001, respectively) but no such differences were observed at 1.5 T (all *p* > 0.05).

### Transmural T_1_ mapping profiles of MOLLI and SAPPHIRE variants

**Fig. 8:**
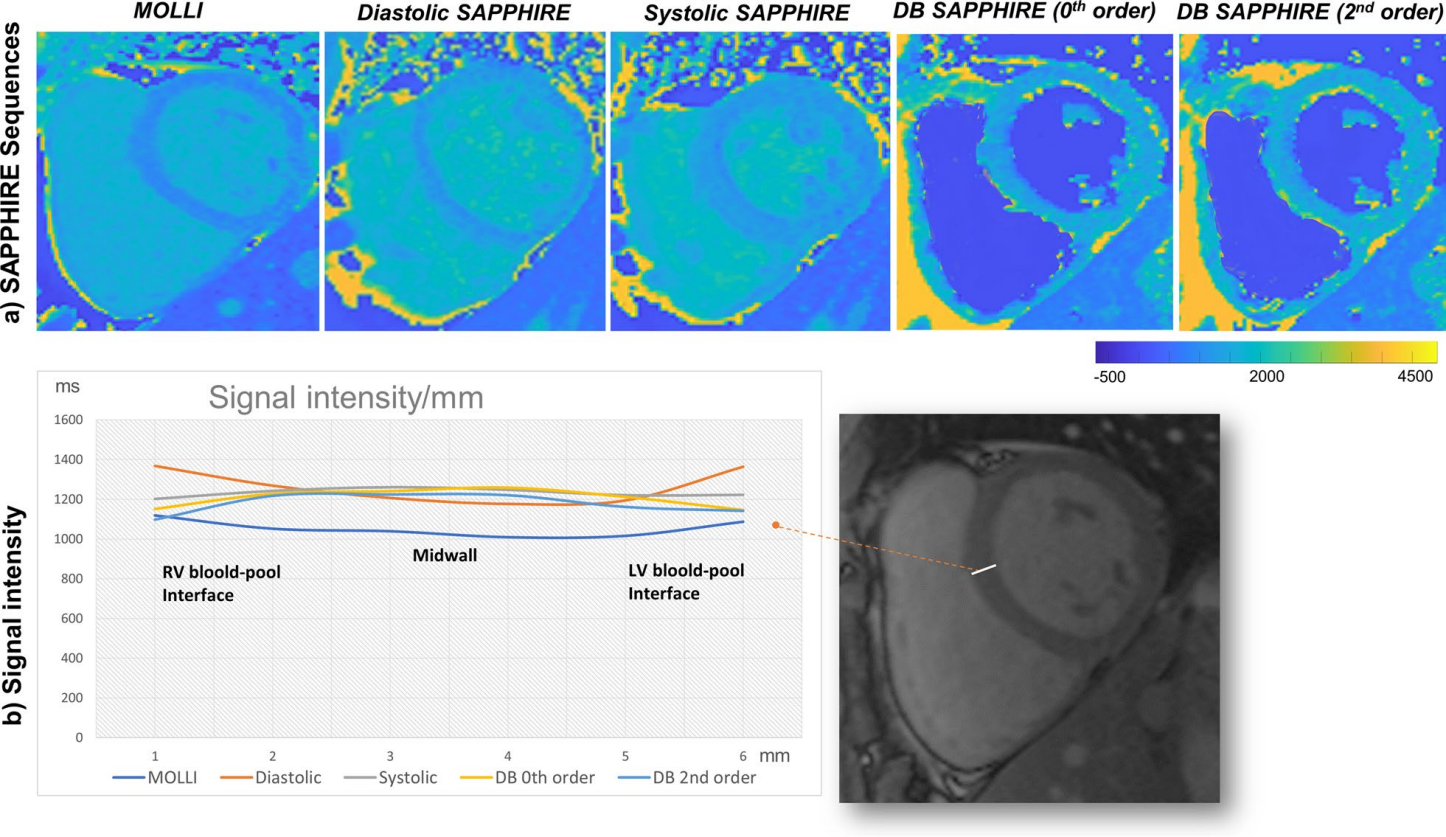
(**a**) Exemplar mid left ventricular short axis slice native T_1_ maps from the same participant by MOLLI and the 4 SAPPHIRE variants (diastolic, systolic, DB 0th and 2nd order) at 1.5 T. (**b**) Averaged transmural 6-point linear profiles (from the RV to LV side of the mid septum) of myocardial T_1_ times for all participants. *LV, left ventricle.* Other abbreviations as in Figs. 1 and 5

Figure 8a shows native T1 maps from MOLLI and the 4 SAPPHIRE variants. Transmural T_1_ times (Fig. 8b) in the mid septum immediately adjacent to the LV and RV blood pools were longer by MOLLI and diastolic SAPPHIRE when compared to systolic, 0th order DB and 2nd order DB SAPPHIRE. Contamination from the high T_1_ of the blood pool appeared as an upsloping T_1_ profile near the edges of the septal profile for both MOLLI and diastolic SAPPHIRE sequences, but not for the other SAPPHIRE variants. Indeed, the profile distributions of transmural myocardial T_1_ times by the Anderson–Darling test differed significantly between MOLLI and all 4 SAPPHIRE variants (diastolic *p* = 0.0003; systolic *p* = 0.0003; 0th order DB *p* = 0.024; 2nd order DB *p* = 0.025); between diastolic SAPPHIRE and both DB variants (0th order DB *p* = 0.0003; 2nd order DB *p* = 0.001); and between systolic SAPPHIRE and both DB variants (0th order DB *p* = 0.0003; 2nd order DB *p* = 0.0005), Furthermore, the dispersion of transmural T_1_ times across the mid septum for systolic (1233 ± 22 ms; interquartile range [IQR] = 25.3 ms) and DB SAPPHIRE variants (0th order: 1206 ± 46 ms; IQR = 71.0 ms | 2nd order: 1178 ± 52 ms; IQR = 72.9 ms) was narrower when compared to diastolic SAPPHIRE (1263 ± 86 ms; IQR = 142.8 ms), and the dispersion for systolic SAPPHIRE narrower than of MOLLI (1054 ± 42 ms; IQR = 56.4 ms).

### Re-read variability of T_1_ mapping measurements

Intra- and interobserver variability of myocardial T_1_ reads was excellent across all sequences tested with ICCs ranging from 0.953–0.996 (Table 5).

**Table 5:** Re-read variability of T_1_ mapping measurements

Sequence	Region	Intra-observer ICC (95% CI)	Inter-observer ICC (95% CI)
MOLLI	Average mid	0.989 (0.91–0.99)	0.968 (0.76–0.99)
	Septal ROI	0.978 (0.78–0.99)	0.950 (0.53–0.99)
Diastolic SAPPHIRE	Average mid	0.996 (0.45–0.99)	0.993 (082–0.99)
	Septal ROI	0.987 (0.89–0.99)	0.954 (0.52–0.99)
Systolic SAPPHIRE	Average mid	0.957 (0.64–0.99)	0.953 (0.54–0.99)
	Septal ROI	0.964 (0.66–0.99)	0.982 (0.87–0.99)
0th order DB SAPPHIRE	Average mid	0.994 (0.95–0.99)	0.984 (0.50–0.99)
	Septal ROI	0.991 (0.76–0.99)	0.986 (0.84–0.99)
2nd order DB SAPPHIRE	Average mid	0.973 (0.78–0.99)	0.967 (0.47–0.99)
	Septal ROI	0.994 (0.79–0.99)	0.992 (0.88–0.99)

Agreement was considered excellent when ICC > 0.74, good when ICC = 0.60–0.74, fair when ICC = 0.40–0.59 and poor when ICC < 0.4

CI, confidence interval; ICC, intraclass correlation coefficient. Other abbreviations as in Tables 1 and 2

## Discussion

This study assessed the feasibility of biventricular SAPPHIRE T1 mapping in vivo across field strengths. We found that native T_1_ was significantly shorter by the systolic, 0th order DB and 2nd order DB SAPPHIRE variants compared to diastolic SAPPHIRE across field strengths. We show that systolic and DB SAPPHIRE variants reduce the dispersion of trans-myocardial T_1_ times across the septum and abolish the artefactual T_1_ lengthening in voxels located at the blood-myocardial/epicardial boundaries that remains a visible problem for both MOLLI and diastolic SAPPHIRE. Combined, these data suggest that systolic and DB SAPPHIRE approaches may help counteract the problem of partial volume effects. According to these data, the 0th and 2nd order DB SAPPHIRE sequences appear to be equivalent at preventing myocardial T_1_ signal contamination by the adjacent blood pool. Systolic SAPPHIRE was the sequence that produced the narrowest dispersion of myocardial T_1_ times across the mid septum.

Previous work at 3 T has suggested that native myocardial T_1_ in health lengthens progressively from base to apex [21, 22] and we observed a similar trend by MOLLI at 3 T but not at 1.5 T, and not with the majority of SAPPHIRE variants used in this study. The most likely explanation for the lengthening T_1_ from base to apex is that partial volume effects are more prevalent in the thinner more apical segments, and exacerbated by the natural curvature of the apical cap.

Results from linear mixed model analysis showing that native T_1_ was longer by diastolic than systolic SAPPHIRE at both field strengths, are in agreement with previous work [9, 11, 23, 24]. It has been postulated that in systolic T_1_ mapping these differences were due to reduced partial volume effects thanks to the increased LV wall thickness. The fact that 0th order and 2nd order DB SAPPHIRE T_1_ times were significantly shorter than diastolic SAPPHIRE suggests that blood pool suppression is another potential solution for the problem of partial volume effects.

We observed that the RV free wall was visible and indeed analysable on the short axis cine slices by systolic and DB SAPPHIRE approaches but hardly visible at all by conventional bright blood SAPPHIRE or MOLLI. We found that RV T_1_ was significantly longer than LV T_1_ which might be due to residual partial voluming and/or the naturally higher collagen content of the RV [25]. The published literature provides conflicting data on the relationship between RV and LV T_1_, with groups reporting higher RV T_1_ [26–28], lower RV T_1_[29] or equivalent RV/LV T_1_ reads [30] in various cohorts, not all of which were healthy controls as in our case. Although we took care to draw precise ROIs in the RV myocardium, partial voluming and contamination of RV T_1_ times from the inadvertent inclusion of voxels contaminated by blood pool or epicardial fat signals are plausible pitfalls that could have artefactually lengthened our native RV myocardial T_1_ times.

Overall, the evidence presented in this study suggests that systolic and DB approaches are less prone to partial volume effects than MOLLI and diastolic SAPPHIRE. The transeptal myocardial T_1_ times immediately adjacent to LV/RV blood pools recorded for 0th and 2nd order DB SAPPHIRE were significantly lower than those recorded for systolic SAPPHIRE (Fig. 8b) suggesting that blood pool nulling achieved by the DB T_1_ mapping approaches provides some additional benefit thanks to the reduced sensitivity to partial volume effects. Future work should continue to explore the potential clinical utility of DB SAPPHIRE T_1_ mapping for the study of thin-walled cardiac structures, such as the RV free wall, atria an apical slices.

We report excellent intra- and inter-observer re-read reproducibility for all the methods studied with all ICCs (> 0.90) being well in line with previous reports [8, 21, 23, 24, 31–34].

In this study we also demonstrate the use of higher order gradient moment nulling for dark blood T_1_ mapping. In previous literature 0th order nulling was found to be susceptible to residual myocardial motion in some cases, necessitating fine tuning of the motion sensitising gradient moment. As the residual cardiac motion is less turbulent compared to blood flow, stronger motion sensitizing gradients may be applied when nulling the gradient moment up to the 2nd order. Even though quantification results were comparable between 0th and 2nd order DB SAPPHIRE in our experiments, 2nd order nulling may have the advantage of increased ease of use for clinical translation. Robustness of higher order gradient nulling for DB T_1_ mapping, thus, warrants further investigation in a patient cohort.

## Limitatons

This small-scale single-center, single-vendor feasibilty study include small numbrer of healthy volunteers, the results hold promise to overcome some conventional T1 mapping limitations. Further work with larger cohorts in health and disease are needed to explore the robustness and validation. The establishment of normal values for T1 by SAPPHIRE was beyond the scope of this study.

Comparison to other T_1_ mapping sequences besides MOLLI was not undertaken and neither was post-contrast T_1_ mapping. Future work will need to examine the clinical utility of these SAPPHIRE sequences in patients with arrhythmia, cardiomyopathy and for the study of other thin-walled cardiac structures such as the atria. RV T_1_ analysis was not possible on MOLLI or diastolic SAPPHIRE due to the RV thickness limitation. MOCO was used for MOLLI sequences but not for the SAPPHIRE T_1_ mapping sequences. Future work should seek to assess the impact of heart rate (HR) variability on the spin dynamic in the systolic SAPPHIRE sequence. This study was not specifically designed to examine the impact of HR on the quality of systolic data, nevertheless we had a broad range of resting HRs across study members (from 56 to 89 bpm). We observed good quality raw systolic images and reconstructed maps regardless of HR (Additional file 1: Figure S1).

Further testing and validation of the systolic SAPPHIRE sequence will be needed to understand its robustness in relation to RV T1 mapping, but our preliminary findings at least indicate that systolic SAPPHIRE T1 mapping permits placement of a decent-sized ROI in the RV free wall, otherwise impossible with conventional T1 mapping approaches.

## Conclusion

These small-scale preliminary healthy volunteer data suggest that DB SAPPHIRE has the potential to reduce partial volume effects at the blood-myocardial interface, and that systolic SAPPHIRE could be a feasible solution for right ventricular T_1_ mapping. Further work is needed to understand the robustness of these sequences and their potential clinical utility.

## Supplementary Information

**Additional file 1**. Saturation-Pulse Prepared Heart-rate independent Inversion-REcovery (SAPPHIRE) Biventricular T1 Mapping: Inter-Field Strength, Head-To-Head Comparison of Diastolic, Systolic and Dark-Blood Measurements. **Table S1**. Pairwise comparison of global T1 values across sequences (including apical slice data). **Table S2**. Pairwise comparison of global T1 values across sequences after excluding the apical slice. **Table 3**. Pairwise comparison of RV T1 values across sequences. **Figure s1**. Systolic SAPPHIRE T1 mapping across a range of resting heart rates.

## Data Availability

The datasets used and/or analysed during the current study are available from the corresponding author on reasonable request.

## Abbreviations

BSA: Body surface area
bSSFP: Balanced steady-state free precession
CI: Confidence interval
CMR: Cardiovascular magnetic resonance
CVI42: Circle Cardiovascular Imaging 42
DB: Dark-blood
ECG: Electrocardiogram
FA: Flip angle
HR: Heart rate
ICC: Intraclass correlation coefficient
LAVi: Left atrial volume indexed to body surface area
LV: Left ventricle
LVEDVi: Left ventricular end-diastolic volume indexed to body surface area
LVEF: LV ejection fraction
LVESVi: Left ventricular end-systolic volume indexed to body surface area
LVMi: Left ventricular mass indexed to body surface area
MAPSE: Mitral annular plane systolic excursion
MOCO: Motion correction
MOLLI: Modified look-locker inversion recovery
MSDE: Motion sensitized driven equilibrium
MWT: Maximum wall thickness
NIHR RD-TRC: National institute for health research rare diseases translational research collaboration
RAVi: Right atrial volume indexed to body surface area
ROI: Region of interest
RV: Right ventricle
RVEDVi: Right ventricular end-diastolic volume indexed to body surface area
RVEF: Right ventricular ejection fraction
RVESVi: Right ventricular end-systolic volume indexed to body surface area
SAPPHIRE: Saturation-pulse prepared heart-rate independent inversion-recovery
SAX: Short axis
SD: Standard deviation
T: Tesla
TAPSE: Tricuspid annular plane systolic excursion
TD: Trigger delay
TI: Inversion time
TR/TE: Time between two consecutive excitations/echo time
UCL: University College London
