# Supplementary material for: Saturation-pulse prepared heart-rate independent inversion-recovery (SAPPHIRE) biventricular T1 mapping: inter-field strength, head-to-head comparison of diastolic, systolic and dark-blood measurements
Source: BMC Med Imaging. 2022 Jul 7;22:122. doi: 10.1186/s12880-022-00843-0 (PMC9264718; doi:10.1186/s12880-022-00843-0)
Supplement: Supplementary file 1 — Additional file 1. Saturation-Pulse Prepared Heart-rate independent Inversion-REcovery (SAPPHIRE) Biventricular T1 Mapping: Inter-Field Strength, Head-To-Head Comparison of Diastolic, Systolic and Dark-Blood Measurements. Table S1. Pairwise comparison of global T1 values across sequences (including apical slice data). Table S2. Pairwise comparison of global T1 values across sequences after excluding the apical slice. Table 3. Pairwise comparison of RV T1 values across sequences. Figure s1. Systolic SAPPHIRE T1 mapping across a range of resting heart rates. [file 12880_2022_843_MOESM1_ESM.docx]

SUPPLEMENTARY MATERIAL FOR:

Saturation-Pulse Prepared Heart-rate independent Inversion-REcovery (SAPPHIRE) Biventricular T1 Mapping: Inter-Field Strength, Head-To-Head Comparison of Diastolic, Systolic and Dark-Blood Measurements

Mashael Alfarih MSc,^1,2,3^ João B Augusto MD,^1,2^ Kristopher D Knott MBBS, ^2^ Nasri Fatih MSc,^2^ Praveen Kumar-M MD,^4^ Redha Boubertakh PhD,^5^ Alun D Hughes MDBS PhD,^2,6^ James C Moon MD MDBS FRCP,^1,2^ Sebastian Weingärtner PhD,^7,8^ Gabriella Captur MD PhD MRCP MSc. ^1,2,6,9^

1. Barts Heart Center, The Cardiovascular Magnetic Resonance Imaging Unit, St Bartholomew’s Hospital, West Smithfield, London, EC1A 7BE, UK
2. Institute of Cardiovascular Science, University College London, Gower Street, London, WC1E 6BT, UK
3. Department of Cardiac Technology, College of Applied Medial Sciences, Imam Abdulrahman Bin Faisal University, Dammam, Saudi Arabia
4. Department of Pharmacology, Post Graduate Institute of Medical Education and Research, Chandigarh, India
5. William Harvey Research Institute, Queen Mary University of London, Charterhouse Square, London, United Kingdom
6. UCL MRC Unit for Lifelong Health and Ageing, 33 Bedford Place, London WC1B 5JU, UK
7. Electrical and Computer Engineering, University of Minnesota, Minneapolis, MN, United States
8. Department of Imaging Physics, Delft University of Technology, Delft, The Netherlands
9. Cardiology Department, Royal Free Hospital NHS Trust, Pond St, Hampstead, London, NW3 2QG, UK

Corresponding Author:

Dr Gabriella Captur

Institute of Cardiovascular Science,

University College London,

Gower Street,

London WC1E 6BT, UK

E-mail: [gabriella.captur@ucl.ac.uk](mailto:gabriella.captur.11@ucl.ac.uk)

Tel: + 44 7809621264

**INDEX FOR SUPPLEMENTARY MATERIAL**

**Supplementary Table 1.** Pairwise comparison of global T1 values across sequences (including apical slice data).

**Supplementary Table 2.** Pairwise comparison of global T1 values across sequences after excluding the apical slice.

**Supplementary Table 3.** Pairwise comparison of RV T1 values across sequences.

**Supplementary Figure 1.** Systolic SAPPHIRE T1 mapping across a range of resting heart rates.

| Pairwise comparisons | | Field strength | Mean difference | SE | *p* value | 95% Confidence interval | |
| --- | --- | --- | --- | --- | --- | --- | --- |
|  |  |  |  |  |  | Lower bound | Upper bound |
| MOLLI *vs.* | Diastolic SAPPHIRE | 1.5T | -249.4 | 12.5 | .000 | -286.4 | -212.5 |
|  |  | 3T | -302.5 | 13.9 | .000 | -343.4 | -261.5 |
|  | Systolic SAPPHIRE | 1.5T | -204.5 | 12.5 | .000 | -241.5 | -167.6 |
|  |  | 3T | -297.5 | 13.5 | .000 | -337.5 | -257.7 |
|  | 0^th^ Order DB SAPPHIRE | 1.5T | -206.8 | 12.5 | .000 | -243.8 | -169.8 |
|  |  | 3T | -298.2 | 13.5 | .000 | -338.1 | -258.4 |
|  | 2^nd^ Order DB SAPPHIRE | 1.5T | -217.1 | 12.5 | .000 | -254 | -180.1 |
|  |  | 3T | -262.5 | 13.5 | .000 | -302.3 | -222.6 |
| 0^th^ Order DB SAPPHIRE *vs.* | Diastolic SAPPHIRE | 1.5T | -42.6 | 12.5 | .014 | -79.5 | -5.7 |
|  |  | 3T | -4.3 | 13.9 | 1.000 | -45.2 | 36.7 |
|  | Systolic SAPPHIRE | 1.5T | 2.3 | 12.5 | 1.000 | -34.6 | 39.3 |
|  |  | 3T | .65 | 13.5 | 1.000 | -39.2 | 40.5 |
|  | 2^nd^ Order DB SAPPHIRE | 1.5T | -10.3 | 12.5 | 1.000 | -47.2 | 26.7 |
|  |  | 3T | 35.7 | 13.5 | .112 | -4.1 | 75.6 |
| 2^nd^ Order DB SAPPHIRE *vs.* | Diastolic SAPPHIRE | 1.5T | -32.3 | 12.5 | .130 | -69.3 | 4.6 |
|  |  | 3T | -40.1 | 13.9 | .060 | -80 | .95 |
|  | Systolic SAPPHIRE | 1.5T | 12.6 | 12.5 | 1.000 | -24.4 | 49.5 |
|  |  | 3T | -35.1 | 13.5 | .126 | -74 | 4.8 |
| Systolic SAPPHIRE *vs.* | Diastolic SAPPHIRE | 1.5T | -2.3 | 12.5 | 1.000 | -39.3 | 34.6 |
|  |  | 3T | -4.9 | 13.9 | 1.000 | -45.9 | 36 |
| DB, dark blood; MOLLI, modified Look-Locker inversion recovery; ROI, region of interest; SAPPHIRE, SAturation Pulse Prepared Heart-rate independent Inversion REcovery; SE, standard error; T, tesla  * The mean difference is significant at the .05 level. | | | | | | | |

**Table 1.** Pairwise comparison of global T_1_ values across sequences (including apical slice data).

**Table 2.** Pairwise comparison of global T_1_ values across sequences after excluding the apical slice.

| Pairwise comparisons | | Field strength | Mean difference | SE | *p* value | 95% Confidence interval | |
| --- | --- | --- | --- | --- | --- | --- | --- |
|  |  |  |  |  |  | Lower bound | Upper bound |
| MOLLI *vs.* | Diastolic SAPPHIRE | 1.5T | -232.8 | 16.8 | .000 | -282.3 | -183.3 |
|  |  | 3T | -316.8 | 19.5 | .000 | -374.4 | -259.2 |
|  | Systolic SAPPHIRE | 1.5T | -221.5 | 16.8 | .000 | -271 | -172 |
|  |  | 3T | -295.2 | 19 | .000 | -351.3 | -239.2 |
|  | 0^th^ Order DB SAPPHIRE | 1.5T | -211.1 | 16.8 | .000 | -260.6 | -161.7 |
|  |  | 3T | -288.3 | 19 | .000 | -344.4 | -232.2 |
|  | 2^nd^ Order DB SAPPHIRE | 1.5T | -205.9 | 16.8 | .000 | -255.4 | -156.4 |
|  |  | 3T | -265.1 | 19 | .000 | -321.1 | -209 |
| 0^th^ Order DB SAPPHIRE *vs.* | Diastolic SAPPHIRE | 1.5T | -21.7 | 16.8 | 1.000 | -71.1 | 27.8 |
|  |  | 3T | -28.5 | 19.5 | 1.000 | -86.1 | 29.1 |
|  | Systolic SAPPHIRE | 1.5T | -10.4 | 16.8 | 1.000 | -59.8 | 39.1 |
|  |  | 3T | -6.9 | 19 | 1.000 | -63.1 | 49.1 |
|  | 2^nd^ Order DB SAPPHIRE | 1.5T | 5.2 | 16.8 | 1.000 | -44.2 | 54.7 |
|  |  | 3T | 23.2 | 19 | 1.000 | -32.8 | 79.3 |
| 2^nd^ Order DB SAPPHIRE *vs.* | Diastolic SAPPHIRE | 1.5T | -26.9 | 16.8 | 1.000 | -76.4 | 22.6 |
|  |  | 3T | -51.7 | 19.5 | .110 | -109.3 | 5.9 |
|  | Systolic SAPPHIRE | 1.5T | -15.6 | 16.8 | 1.000 | -65.1 | 33.9 |
|  |  | 3T | -30.2 | 19 | 1.000 | -86.2 | 25.9 |
| Systolic SAPPHIRE *vs.* | Diastolic SAPPHIRE | 1.5T | -11.3 | 16.8 | 1.000 | -60.8 | 38.1 |
|  |  | 3T | -21.6 | 19.5 | 1.000 | -79.2 | 36 |
| DB, dark blood; MOLLI, modified Look-Locker inversion recovery; ROI, region of interest; SAPPHIRE, SAturation Pulse Prepared Heart-rate independent Inversion REcovery; SE, standard error; T, tesla  * The mean difference is significant at the .05 level. | | | | | | | |

**Table 3.** Pairwise comparison of RV T_1_ values across sequences

| Pairwise comparisons | | Field strength | Mean difference | SE | *p* value | 95% Confidence interval | |
| --- | --- | --- | --- | --- | --- | --- | --- |
|  |  |  |  |  |  | Lower bound | Upper bound |
| Systolic *vs.* | 0^th^ Order DB SAPPHIRE | 1.5T | 29.2 | 31.4 | 1.000 | -48.7 | 107.1 |
|  |  | 3T | 65.5* | 24.3 | .028 | 5.4 | 125.6 |
|  | 2^nd^ Order DB SAPPHIRE | 1.5T | 16.4 | 31.4 | 1.000 | -61.4 | 94.3 |
|  |  | 3T | 93.2* | 23.7 | .001 | 34.6 | 151.9 |
| 0^th^ Order DB SAPPHIRE *vs.* | 2^nd^ Order DB SAPPHIRE | 1.5T | -12.8 | 32.3 | 1.000 | -92.8 | 67.2 |
|  |  | 3T | 27.7 | 20.3 | .536 | -22.6 | 78.0 |
| DB, dark blood; MOLLI, modified Look-Locker inversion recovery; ROI, region of interest; SAPPHIRE, SAturation Pulse Prepared Heart-rate independent Inversion REcovery; SE, standard error; T, tesla  * The mean difference is significant at the .05 level. | | | | | | | |


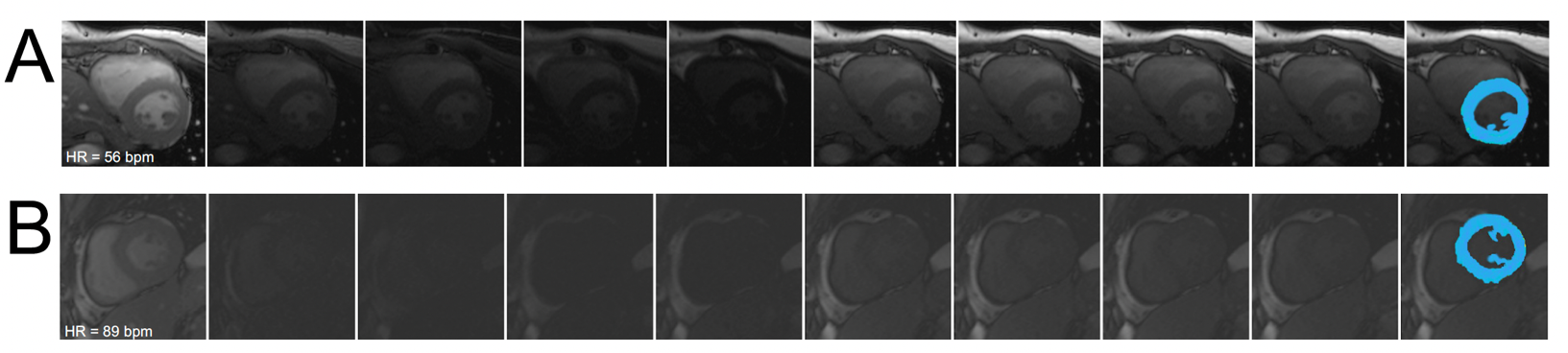
**Supplementary Figure 1.** Systolic SAPPHIRE T1 mapping across a range of resting heart rates. Series of T1 weighted baseline images with different inversion times and the corresponding T1 map overlay as acquired in the systolic SAPPHIRE T1 mapping sequence across a range of heart rates in study members: (A) systolic T1 mapping in a healthy subject with ow resting heart rate of 56pm; (B) healthy subject with higher resting heart rate of 89bpm.
